# Sublingual Dermoid Cyst Mimicking Ranula on Imaging: Surgical Treatment and Outcome in a Human Immunodeficiency Virus (HIV)-Positive Patient

**DOI:** 10.7759/cureus.85534

**Published:** 2025-06-07

**Authors:** Gulistan Bano, Dheeraj Kumar, Swaha Panda, Bhartendu Bharti, Pradosh Kumar Sarangi

**Affiliations:** 1 Otolaryngology-Head and Neck Surgery, All India Institute of Medical Sciences, Deoghar, Deoghar, IND; 2 Radiodiagnosis, All India Institute of Medical Sciences, Deoghar, Deoghar, IND

**Keywords:** congenital dermoid cyst, congenital epidermoid cyst, dysphagia, extraoral excision, hiv-positive, midline neck mass, oral ranula, sublingual lesion

## Abstract

Dermoid cysts are rare congenital lesions that arise due to the entrapment of ectodermal and mesodermal elements during embryonic development. We report the case of a 21-year-old male with HIV who presented with a longstanding sublingual mass associated with difficulty in chewing and swallowing. Clinical examination revealed a firm, non-tender, and mobile sublingual mass, accompanied by concurrent submental swelling. Ultrasonography and computed tomography of the neck were suggestive of ranula. The patient underwent surgical excision through an extraoral submental approach under general anesthesia. The cyst was found to be attached to the mylohyoid muscle and was carefully dissected and removed in its entirety. Histopathological analysis confirmed a dermoid cyst, showing stratified squamous epithelium lined by keratin and sebaceous glands. The patient's postoperative recovery was smooth, with no recurrence noted at the three-month follow-up. Although rare, dermoid cysts of the oral cavity should be considered in the differential diagnosis of midline sublingual swellings. Imaging modalities, including ultrasonography and computed tomography (CT), are integral for preoperative assessment, although they may occasionally present challenges in achieving a definitive diagnosis. Complete surgical excision is the definitive treatment, with the decision to use either an intraoral or extraoral approach based on the size and location of the cyst and the risk of infection. Early detection and treatment are vital to avoid complications like airway obstruction and difficulty swallowing.

## Introduction

Dermoid cysts are congenital developmental anomalies that arise from the sequestration of ectodermal, mesodermal, or endodermal tissues within otherwise normal anatomical structures. These anomalies result from an incomplete fusion of the embryonic lateral mesenchymal processes, typically occurring during the fifth week of embryogenesis [1].

Both epidermoid and dermoid cysts are benign neoplasms that can develop in various regions of the body, with approximately 7% localizing to the head and neck region and around 1.6% manifesting within the oral cavity [2,3].

These cysts generally exhibit slow, progressive growth and, although present at birth, often become clinically apparent during the second or third decades of life. The recommended management is complete surgical excision, with the choice between an intraoral or extraoral approach determined by the cyst’s size and anatomical position [2-4].

## Case presentation

A 21-year-old male patient presented to the otolaryngology outpatient department in a tertiary health care center in Eastern India with a bulge on the floor of his mouth since childhood. He had difficulty chewing and swallowing solid food more than liquid, which was gradually progressive for the last year. The patient denied experiencing pain, dyspnea, any history of prior surgical interventions, or trauma to the oral cavity or neck. He reported having a known case of the human immunodeficiency virus (HIV) and is currently on the tenofovir disoproxil fumarate, lamivudine, and dolutegravir (TLD) regimen antiretroviral therapy (tenofovir disoproxil fumarate, lamivudine, and dolutegravir) and is doing well.

**Figure 1:**
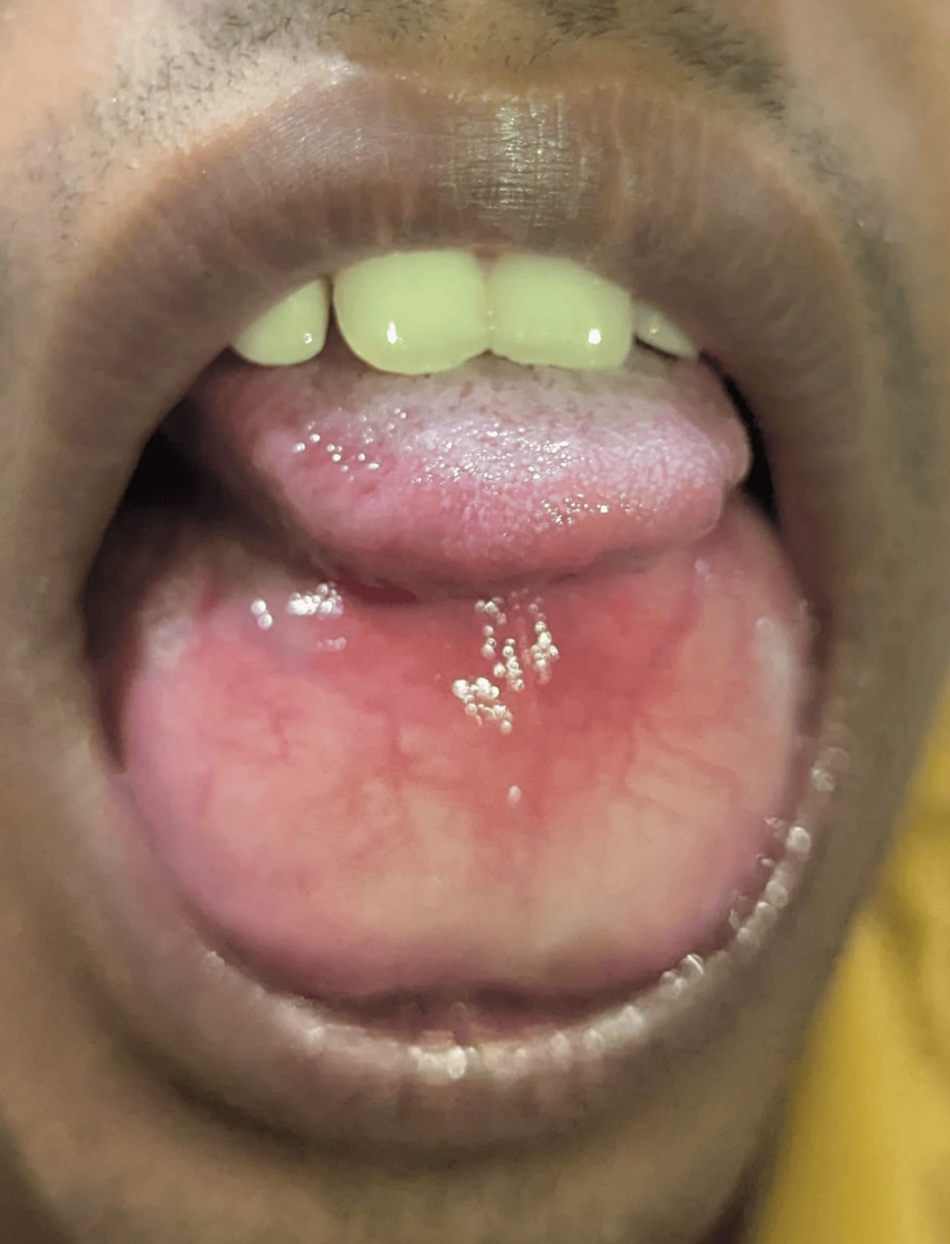
Preoperative clinical presentation showing a dome-shaped, symmetrical swelling in the sublingual region.

Systemic examination did not reveal any abnormalities. Cardiovascular, respiratory, gastrointestinal, and neurological systems were within normal limits, with no signs of organomegaly, lymphadenopathy, cyanosis, clubbing, or edema. Vital parameters were stable. Physical examination of the neck showed a fullness in the submental region. The overlying skin appeared normal, with no evidence of secondary changes, inflammation, or cervical lymphadenopathy. Intraoral examination revealed a solitary, midline, smooth, sublingual swelling measuring approximately 4 × 4 cm pushing the tongue superiorly, as shown in Figure 1. Routine laboratory examinations were within normal limits except for a mild low CD4 count of 468 cells/mm³ (normal range: 500-1500 cells/mm³). His viral load was reported as TND (target not detected).

**Figure 2:**
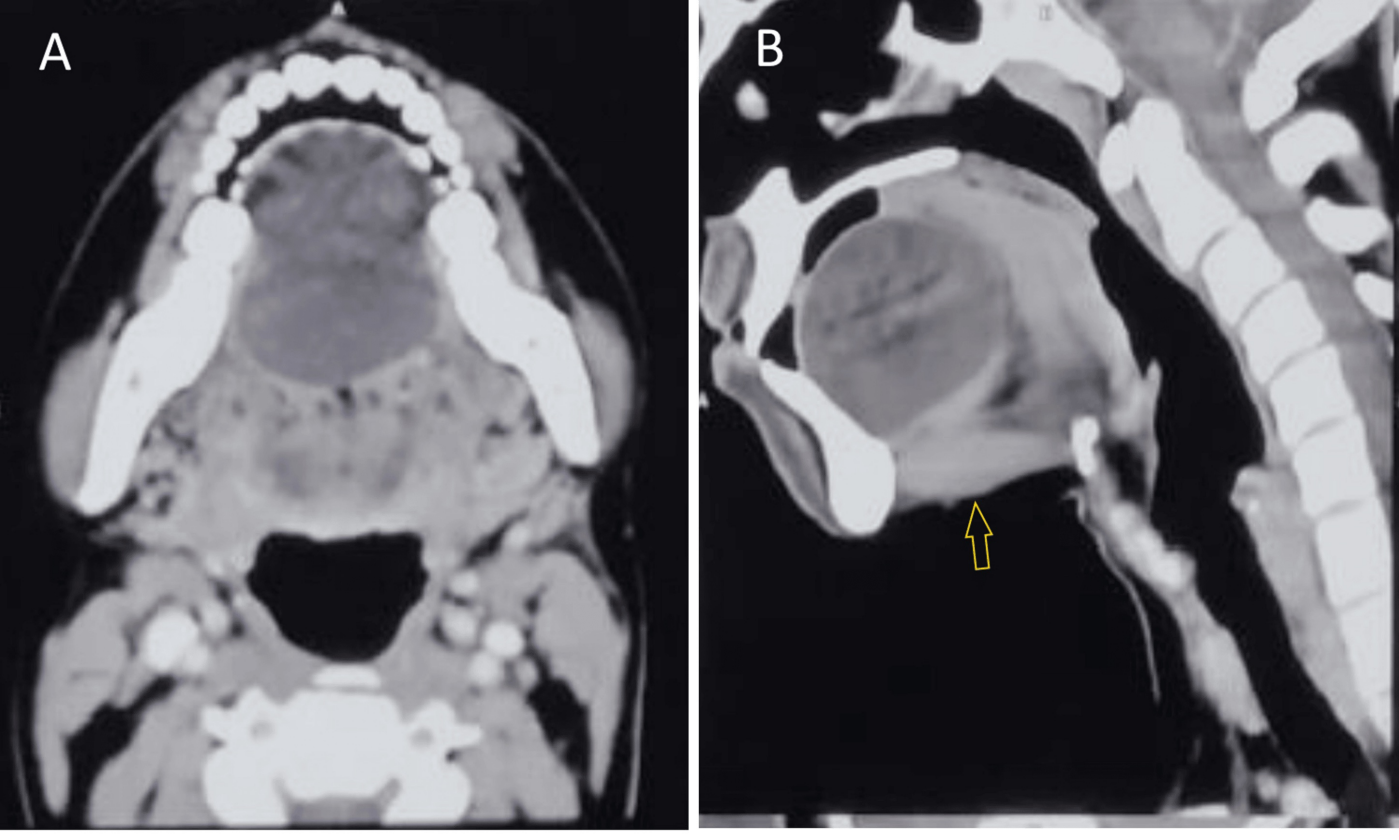
Axial (a) and sagittal (b) contrast-enhanced CT images showing a well-circumscribed cystic lesion in the sublingual space. The arrow denotes the mylohyoid muscle.

Ultrasonography (USG) of the neck was advised and showed a well-circumscribed avascular hypoechoic lesion in the sublingual region, measuring approximately 49 × 48 × 42 mm, containing internal echogenic foci. No fat-fluid levels or echogenic globules were appreciated. The initial differential diagnosis considered was a ranula. Other potential diagnoses, including thyroglossal duct cyst, dermoid cyst, and epidermoid cyst, were also taken into account, as these represent common cystic lesions occurring in the midline of the neck. Contrast-enhanced computed tomography (CECT) of the neck was further advised to assess the size, content, location, and extent of the lesion for preoperative planning (Figures 2A, 2B). It showed a well-defined, non-enhancing cystic lesion in the sublingual space above the mylohyoid muscle, measuring approximately 4.6 × 3.5 × 4.5 cm, displacing the tongue posterosuperiorly. The rest of the CT findings of the neck were unremarkable. A provisional diagnosis of ranula was made, with other benign developmental cysts such as dermoid and epidermoid cysts also taken into consideration. Magnetic resonance imaging (MRI) of the neck was planned to further characterize the lesion to differentiate between dermoid and epidermoid cysts, as epidermoid cysts typically demonstrate diffusion restriction. However, the imaging could not be performed due to financial constraints faced by the patient. The patient was planned for surgical excision of the cyst.

**Figure 3:**
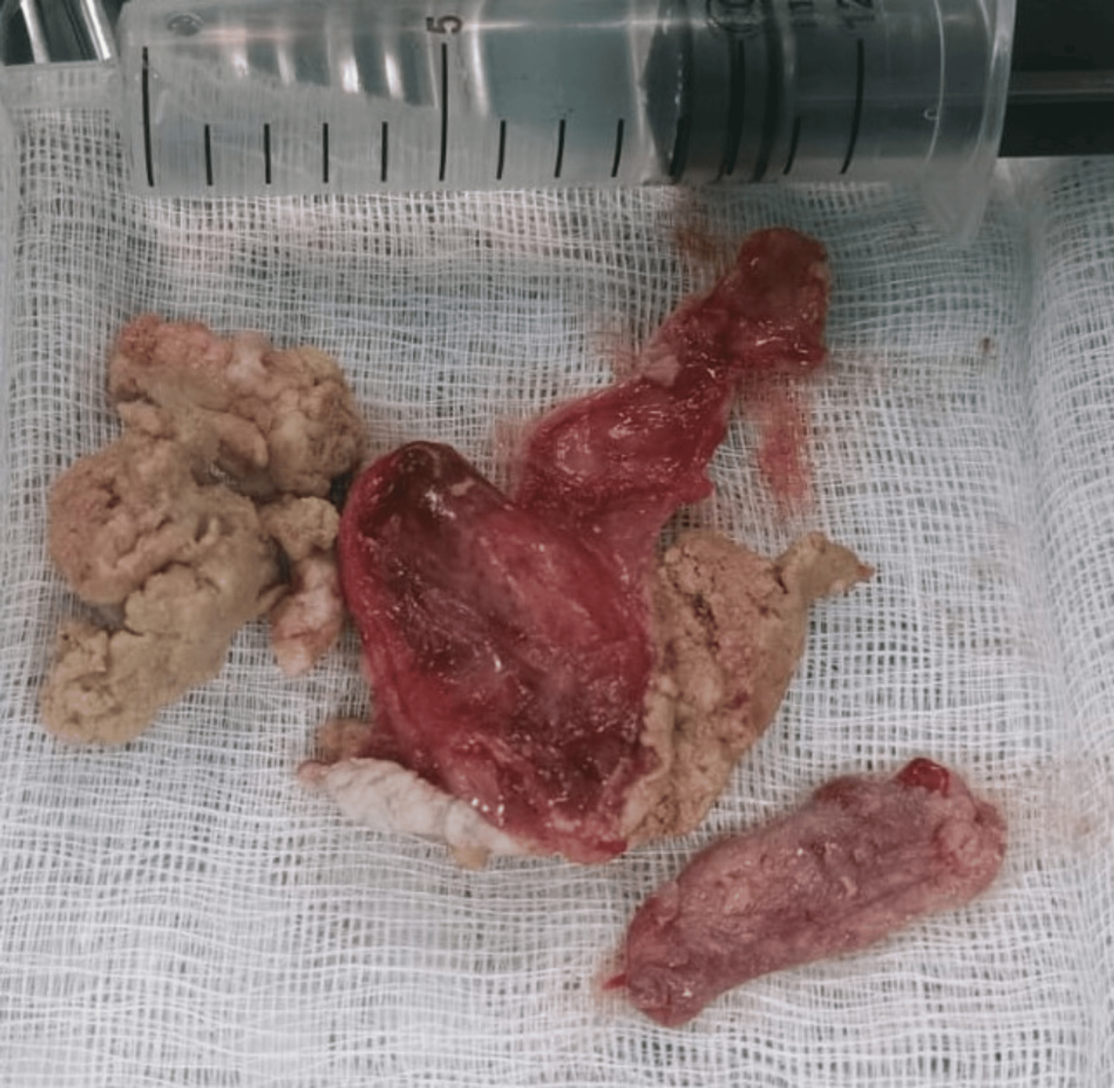
Dissected cyst revealing a cyst wall with cheesy, solid material inside.

The surgery was done under general anesthesia using an external submental approach under all universal precautions since the patient was HIV seropositive. A horizontal midline incision was made along a natural skin crease. The cyst, which was adherent to the mylohyoid muscle, was meticulously dissected and excised in its entirety. During the excision, the cyst ruptured, releasing cheesy paste-like material from the sac (Figure 3). The surgical site was closed in multiple layers, and a non-suction drain was placed for 24 hours. The postoperative period was without complications, and the patient’s tongue regained its anatomical baseline position.

**Figure 4:**
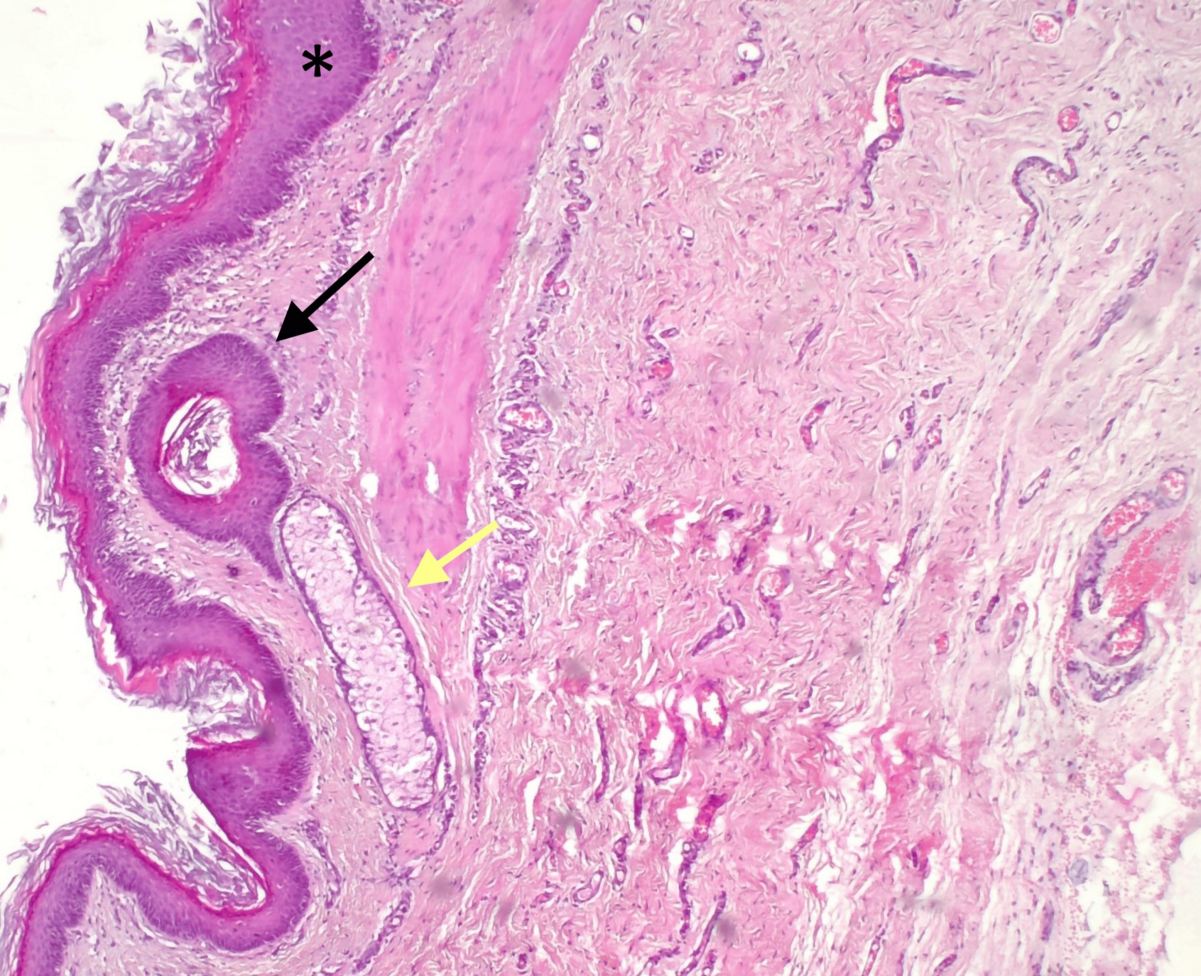
Histopathological examination showing keratinized stratified squamous epithelium (*), hair follicles (black arrow), and sebaceous glands (yellow arrow) (hematoxylin and eosin stain, original magnification ×200).

Histopathological analysis demonstrated that the cyst was lined by stratified squamous epithelium and contained keratinous debris, hair follicles, and sebaceous glands, as illustrated in Figure 4. These findings confirmed the diagnosis of a dermoid cyst.

**Figure 5:**
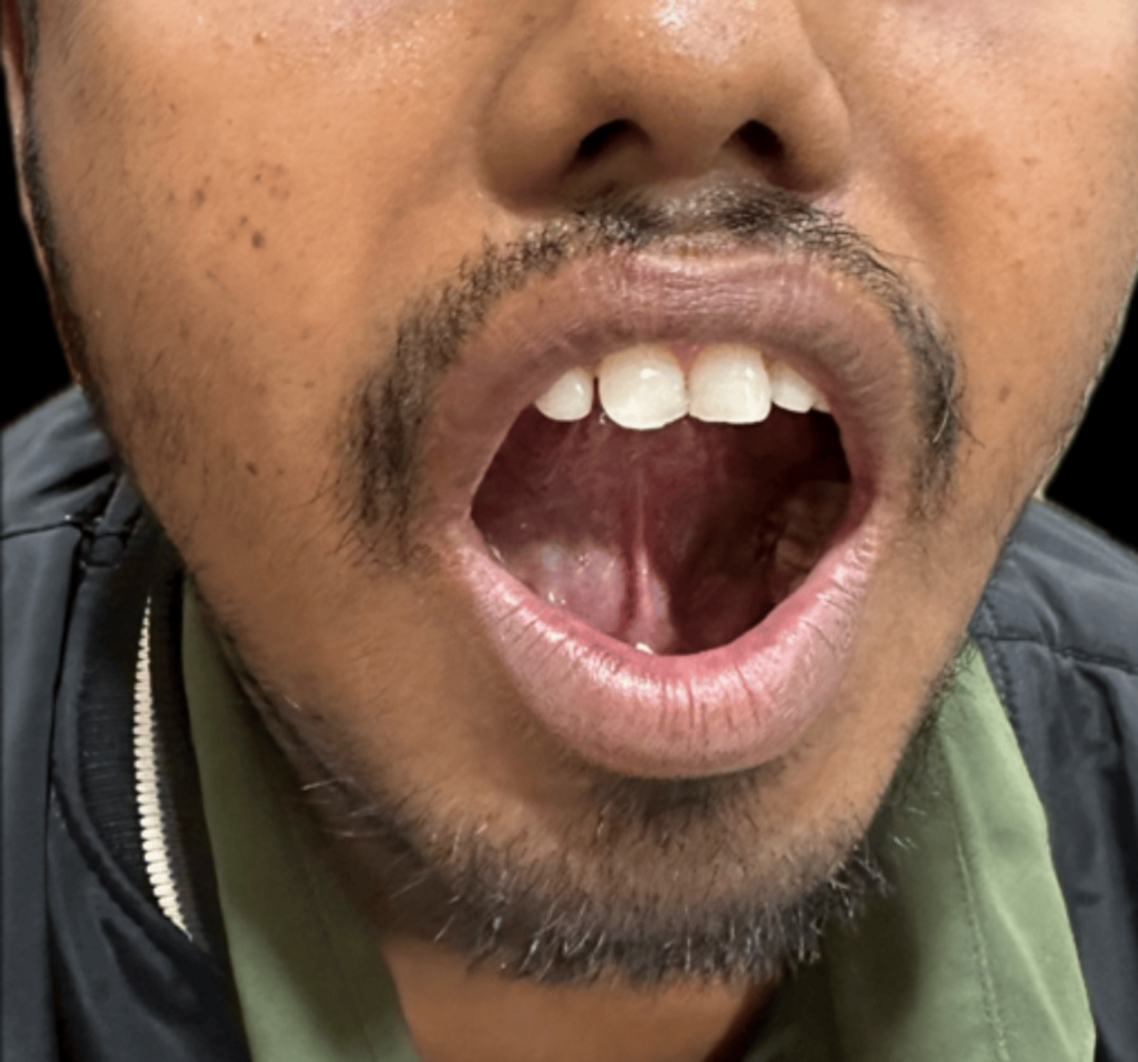
Postoperative clinical photograph at three-month follow-up.

A postoperative follow-up at three months revealed satisfactory healing at the surgical site with no evidence of recurrence or complications (Figure 5).

## Discussion

Dermoid cysts of the floor of the mouth represent developmental anomalies arising from the entrapment and subsequent proliferation of epithelial cells during the midline fusion of the first and second branchial arches, typically occurring between the third and fourth weeks of embryogenesis [1]. A histopathological classification system was given by Meyer in 1955 for these cysts, designating them as epidermoid when lined solely by stratified squamous epithelium, dermoid when skin adnexal structures are present, and teratoid when additional tissues such as muscle, cartilage, or bone are incorporated [5]. Anatomically, dermoid cysts are classified into three categories based on their location: median genio-glossal (supra mylohyoid), median geniohyoid (infra mylohyoid), and lateral. Among all dermoid cysts in the head and neck region, the floor of the mouth represents the second most common site of occurrence, following the lateral eyebrow [4,6]. The most recent classification, proposed by Gordon et al., revised Meyer’s system by introducing the term ‘congenital germ cell fusion cysts’ to characterize these lesions [7].

Sublingual masses can have a broad differential diagnosis, including developmental cysts, infectious etiologies, ranulas, lymphatic malformations, heterotopic gastrointestinal cysts, and foregut duplication cysts [2-7].

Two principal pathogenic mechanisms are recognized: congenital inclusion and acquired implantation. Congenital inclusion cysts arise during embryogenesis due to aberrant fusion of embryonic structures, leading to the sequestration of epithelial elements. Conversely, acquired implantation occurs when epithelial fragments are introduced into deeper tissues, typically following surgical procedures or traumatic events [8,9]. In the present case, there was no history of surgical intervention or trauma, and the swelling had been present since childhood, supporting the likelihood of a congenital inclusion cyst. Moreover, some authors have documented the enlargement of these lesions during puberty, which is attributed to increased sebum production [10].

Radiologic evaluation is essential for the diagnosis and management of dermoid cysts. Ultrasonography typically demonstrates a pseudo-solid echogenic cystic lesion, often displaying a fat-fluid interface or echogenic floating debris, characteristic of its cystic composition [10]. CT commonly reveals a thin-walled, unilocular lesion containing fat, with a dependent fluid level that may shift with changes in patient positioning. However, in this case, the cystic lesion is well-defined with homogenous content. Magnetic resonance imaging (MRI) and ultrasonography offer superior soft tissue contrast relative to computed tomography (CT), thereby providing enhanced delineation of the internal architecture of a mass lesion [11-14]. Magnetic resonance imaging (MRI) shows hyperintense signals on T1-weighted images due to fatty content and signal suppression on fat-saturated sequences. Although both appear as cystic lesions on CT and MRI, dermoid cysts typically show the characteristic feature of coalesced fat globules, often described as resembling a "sac of marbles" [12-14]. Both CT and MRI are valuable for assessing adjacent anatomical structures, aiding in preoperative planning, and surgical approach selection. However, in this case, MRI was not performed due to the patient’s financial constraints. In our case, ultrasound and CT could not differentiate a dermoid cyst from a ranula, which is consistent with previous reports [12,13]. Previous case reports have also described difficulty in differentiating dermoid cysts and ranula even with MRI [12,13]. In the largest retrospective case series of pediatric sublingual dermoid and epidermoid cysts, it was observed that preoperative diagnoses were frequently inconclusive, with approximately 25% of cases being misdiagnosed as ranulas-the most common diagnostic error in this context [15].

Treatment for dermoid cysts usually involves enucleation, with the approach being either intraoral or extraoral. An intraoral surgical approach is generally favored for sublingual cysts smaller than 6 cm and situated above the mylohyoid muscle, while cysts exceeding 6 cm in size are usually treated using an extraoral approach [4,16]. However, in this case, an extraoral surgical approach was selected due to the potential risk of HIV transmission. Although recurrence is uncommon after complete surgical removal, there are reports indicating that up to 5% of oral dermoid cysts may undergo malignant transformation into the teratoid variant [16].

Some authors discovered that internalized stigma was frequently experienced, with over 80% of persons living with HIV agreeing with at least one indicator of stigma, feeling such as “guilty,” “dirty,” “ashamed,” or “worthless” due to their HIV status and worrying about disclosing their serostatus [17]. This might cause more delay in presentation to a clinician, which might cause airway compromise or difficulty in swallowing. Hence, addressing dermoid in such seropositive cases at the earliest is important.

## Conclusions

Dermoid cysts of the floor of the mouth, although rare, should be considered in the differential diagnosis of midline oral cavity masses. Imaging modalities are essential for preoperative assessment but are often inconclusive. Due to their slow-growing nature, these cysts may not present symptoms until later in life, often leading to late diagnoses. The extraoral submental approach provides excellent access for excising larger cysts, ensuring complete removal and minimizing the risk of recurrence and infection in seropositive cases. Given the potential for airway obstruction in severe cases, early surgical intervention is crucial.
